# Micronutrient status during paediatric critical illness: A scoping review

**DOI:** 10.1016/j.clnu.2020.04.015

**Published:** 2020-12

**Authors:** L.V. Marino, F.V. Valla, R.M. Beattie, S.C.A.T. Verbruggen

**Affiliations:** a Department of Dietetics/Speech & Language Therapy, NIHR Biomedical Research Centre Southampton, University Hospital Southampton NHS Foundation Trust and School of Health Sciences, University of Southampton, Southampton, UK; b Paediatric Intensive Care Unit, Hôpital Femme Mère Enfant, CarMEN INSERM UMR 1060 Hospices Civils de Lyon, Lyon-Bron, France; c Department of Paediatric Gastroenterology, Southampton Children's Hospital, NIHR Biomedical Research Centre Southampton, University Hospital Southampton NHS Foundation Trust, Southampton, UK; d Intensive Care, Department of Paediatrics and Paediatric Surgery, Erasmus Medical Centre, Sophia Children's Hospital, Rotterdam, the Netherlands

**Keywords:** Paediatric intensive care, Critically ill children, Micronutrients, Nutrition, Vitamins

## Abstract

**Background:**

No evidence based recommendations for micronutrient requirements during paediatric critical illness are available, other than those arising from recommended nutrient intakes (RNI) for healthy children and expert opinion.

**Objectives:**

The objective of this review is to examine the available evidence from micronutrient status in critically ill children considering studies which describe 1) micronutrient levels, 2) associations between micronutrient levels and clinical outcome, and 3) impact on clinical outcome with micronutrient supplementation during PICU admission.

**Design:**

Scoping review.

**Eligibility criteria:**

Any study which used a qualitative and quantitative design considering causes and consequences of micronutrient levels or micronutrient supplementation during paediatric critical illness.

**Sources of evidence:**

NICE Healthcare Databases Advanced Search website (https://hdas.nice.org.uk/) was used as a tool for multiple searches, with a content analysis and charting of data extracted.

**Results:**

711 records were identified, 35 were included in the review. Studies evaluated serum micronutrient status was determined on admission day in majority of patients. A content analysis identified (n = 49) initial codes, (n = 14) sub-categories and (n = 5) overarching themes during critical illness, which were identified as: i) low levels of micronutrients, ii) causes of aberrant micronutrient levels, iii) associations between micronutrients levels and outcome, iv) supplementation of micronutrients.

**Conclusion:**

During critical illness, micronutrients should be provided in sufficient amounts to meet reference nutrient intakes for age. Although, there is insufficient data to recommend routine supplementations of micronutrients at higher doses during critical illness, the ‘absence of evidence should not imply evidence of absence’, and well designed prospective studies are urgently needed to elucidate paediatric micronutrient requirements during critical illness. The absence of reliable biomarkers make it challenging to determine whether low serum levels are reflective of a true deficiency or as a result redistribution, particularly during the acute phase of critical illness. As more children continue to survive a PICU admission, particularly those with complex diseases micronutrient supplementation research should also be inclusive of the recovery phase following critical illness.

**What we know:**

• There are no evidence based micronutrient requirements during paediatric critical illness.
• Serum levels of micronutrients (vitamins and trace elements) may be affected by critical illness and inflammation.
• There is no evidence that supplementation of micronutrients improves clinical outcomes.

**What this study adds:**

• The results of this scoping review suggest there are numerous gaps in knowledge particularly relating to;
  ○ the interpretation of plasma/serum levels of individual micronutrients during critical illness
  ○ the causality of associations between micronutrient levels and clinical outcomes
  ○ the impact on clinical outcome with micronutrient supplementation during PICU admission
• There is insufficient data to recommend routine supplementations of micronutrients in doses above recommended nutrient intakes levels during critical illness, however, well designed prospective studies are urgently needed to elucidate micronutrient requirements in children during critical illness.

## 1 Introduction

Micronutrients are defined by the World Health Organisation (WHO) *as* “*“magic wands” that enable the body to produce enzymes, hormones and other substances essential for proper growth and development. As tiny as the amounts are, however, the consequences of their absence are severe”* [1]. Micronutrient deficiencies are common amongst children between 6 months and 5 years of age, with 50% being deficient in one or more micronutrients [2].

Children admitted to paediatric intensive care unit (PICU) may have an acute illness or an exacerbation of a complex chronic condition. The median age of children to a paediatric intensive care unit (PICU) is 1.9 years, with the most common indications for admission being respiratory disease, congenital heart disease, and neurologic disorders [3].

Studies investigating the impact of nutritional support on clinical outcome of paediatric critical illness are scarce and have focused on macronutrient (energy and protein) requirements [[4], [5], [6], [7]]. Micronutrient deficiencies have been described in critically ill adults, with low serum levels of thiamine, folate, vitamin B12 and zinc [8]. There is a paucity of clinical data relating to micronutrient requirements in critically ill adults with expert opinions recommending providing micronutrients to dietary reference nutrient intake levels in addition to pharmacological doses of thiamine to prevent refeeding syndrome. Other expert recommendations for adults include higher doses of vitamin C, D, B12, folate, zinc and carnitine to replete low serum levels [[8], [9], [10]]. However, micronutrient deficiencies may precede admission or occur as a result of inadequate intake, acute stress response, increased requirements or excessive losses, all of which may impact on morbidity and compromise clinical outcomes [8,11,12]. Micronutrient pathophysiology and requirements during paediatric critical illness are not well defined, other than those based on reference nutrient intakes (RNI) for healthy children [13] and expert opinion [14,15].

The goal of this scoping review was to systematically assess and describe the published literature for micronutrient requirements in critically ill children, while identifying key themes to guide the development of a conceptual framework for future research with regards to micronutrient status [16]. The objective of the review was to examine the available evidence from micronutrient studies in critically ill children considering those studies which describe 1) micronutrient levels during critical illness, 2) associations between micronutrient levels and clinical outcome, 3) potential causes for low micronutrient levels 4) impact on clinical outcome with micronutrient supplementation during PICU admission.

## 2 Methods

### 2.1 Preparing to scope the literature and protocol development

A scoping review was conducted in order to identify the key concepts that underpin this area of research [16]. The scoping study design was chosen because it offered a framework to identify and synthesize a broad range of evidence. The scoping review methodology provided an opportunity to focus on this complex area of paediatric critical illness and develop a conceptual framework to help identify gaps in the literature and future research priorities [17]. The Preferred Reporting Items for Systematic reviews and Meta-Analyses extension for Scoping Reviews (PRISMA-ScR) was used to develop and report the evidence reviewed for this study [18].

### 2.2 Identifying the research question

• Is it possible to use the available literature to describe micronutrient levels during critical illness and provide recommendations for micronutrient requirements for critically ill children?
• Is there an association with micronutrient status and clinical outcomes in critically ill children?
• What are the current gaps in our knowledge and how could these be addressed?

Using the PRISMA-ScR checklist [18] and other published work [16,17,19] an *a priori* scoping review protocol was developed which included 1) the research question 2) eligibility criteria of the studies be to included, 3) information sources to be searched, 4) description of a full electronic search strategy, 5) data charting process with data items included, 6) critical appraisal and synthesis of the data in order to answer the questions posed. For the purpose of this review, critically ill children were defined as children between the ages of >37 weeks gestational age and 18 years of age in PICU.

### 2.3 Data sources – stage 1

After finalising the objectives and research questions a literature search was completed to identify relevant studies. NICE Healthcare Databases Advanced Search website (https://hdas.nice.org.uk/) was used as a tool for multiple searches within multiple databases including the PsycInfo, Cumulative Index to Nursing and Allied Health Literature (CINAHL) and Medline. PubMed, the Cochrane Library and NHS Evidence were also searched, with searches adapted for each database. Forward and backward citation searching was completed on studies exploring micronutrients in paediatric critical illness, with searches completed until October 2019. The rationale for this time period was to not set a time limit to ensure that as much evidence as possible was captured.

### 2.4 Search strategy – stage 2

A search strategy was devised with the assistance of an information specialist for PubMed using key words from paediatric critical care articles and modified for additional electronic data bases (Supplementary File: Table 1).

**Table 1:** Development of codes, sub-categories and overarching themes.

Initial coding (n = 49)	Sub-categories (n = 20)	Overarching themes/categories (n = 5)
Thiamine Riboflavin Folate Vitamin A β-carotene Ascorbate levels Zinc Selenium Iron Chromium	Excessive losses Inadequate intake Micronutrient malnutrition Insufficiency/deficiency	Low levels of serum/plasma micronutrients [[31], [32], [33], [34], [35], [36], [37], [38], [39], [40], [41]]
Vitamin E Vitamin B6 Copper Molybdenum Niacin Pantothenic acid Biotin Iodine	High levels Unchanged No information available	Serum/plasma micronutrient levels unchanged or high during critical illness [38,[42], [43], [44]]
Low enteral intake Malnutrition Prematurity Congenital heart disease Sepsis/Septic shock Severity of risk scores Inotrope(s) Disease severity Severe pneumonia Lymphopenia Inflammatory response Inflammatory mediators; Interleukin-6 C-reactive protein Fluid restriction Fluid overload/redistribution/dilution Renal replacement therapy Diuretics At risk groups	Phase of illness Inflammatory response Dilution/redistribution Disease process Oxidative stress Medication	Associatiated causes of changes in micronutrients levels [21,22,30,31,37,38,42,[44], [45], [46], [47], [48], [49], [50], [51], [52], [53], [54], [55]]
Mortality risk Duration of mechanical ventilation Development of multi-organ failure Organ dysfunction > 1 Length of PICU stay	Clinical outcomes Impact	Associations between micronutrients levels and morbidity and mortality [40,41,46,53,56]
Selenium Zinc	Safety Outcomes	Supplementation of micronutrients [21,22,46]

### 2.5 Study selection – stage 3

After screening titles and abstracts and deletion of duplicates, full text articles were reviewed for eligibility. Where the same cohort of children were included in multiple articles they were only counted once [[20], [21], [22]]. Inclusion criteria were; any study which used a qualitative and quantitative design considering micronutrient supplementation or characterisation of serum/plasma micronutrient levels during various phases of critical illness in children published in the English language; based on human subjects and published up to October 2019. References from the bibliographies of studies included were hand searched for additional studies which may fulfil the inclusion criteria. Exclusion criteria which were considered not to be in scope of this review included; not English language or that were completed in other healthcare settings (e.g. ward or community environment), children with inherited metabolic disorders and levels described in fluids other than serum/plasma. Micronutrients were defined as chemical element or substance required in trace amounts for the normal growth and development [1,23,24]. Micronutrients excluded were those routinely measured as part of daily clinical biochemical monitoring such as sodium, potassium, calcium, chloride, magnesium, phosphorus [25] vitamin D (as this was classified a hormone) [26], and micronutrient levels not measured using plasma or serum samples [11]. Children with major burns were also excluded from this review as they are not commonly managed within a PICU but a dedicated burns unit, although there may be exceptions to this. In addition, micronutrient status and requirements of this cohort of patients is expertly reviewed within ESPEN endorsed recommendations for nutritional therapy in major burns [27].

### 2.6 Data extraction – stage 4

Data extraction was completed using a two stage process, with a data extraction template (Microsoft 2010, Redmond, WA) used to capture the study design, results and conclusions was created, followed by a content analysis.

### 2.7 Collating, summarizing and reporting the results - stage 5

Data synthesis was completed using an established content analysis approach [28]. Content analysis was chosen as it is a suitable technique for reporting common issues reported in data [29]. Using this approach, the descriptive aspects about the population of interest, methodology, outcomes and any key findings were coded. A content analysis was completed by selecting, coding and creating initial codes, sub-categories and overarching themes/categories. These codes were then grouped into a number of categories and then grouped again into sub-categories. The key overarching categories from this process were developed into a conceptual framework using overarching themes.

## 3 Results

**Fig. 1:**
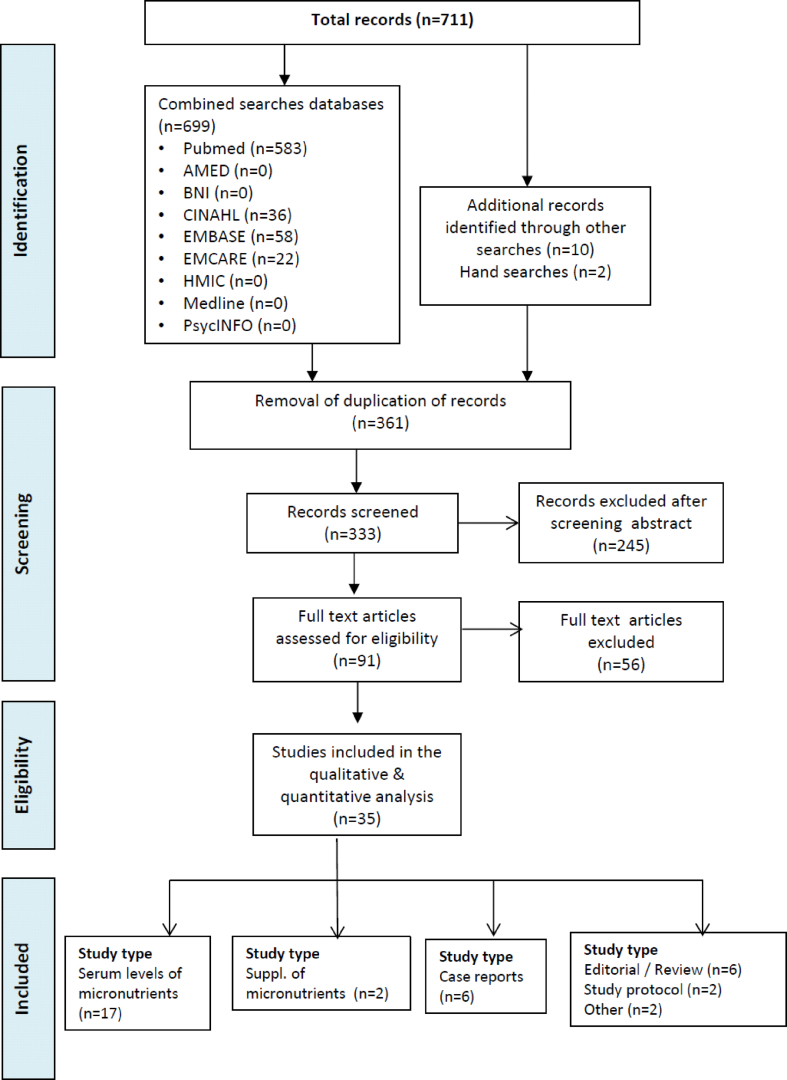
Search results through to inclusion.

711 records were identified, of which 361 were duplicates. Following removal of duplicate records, abstracts and titles of 334 records were screened for inclusion. The full texts of 91 articles were reviewed for eligibility, of which 35 were included in the review (Fig. 1).

### 3.1 Content analysis: conceptual framework and overarching themes

A content analysis identified 49 initial codes, 14 sub-categories and 5 overarching themes (Table 1), which were identified as:

i) Low levels of micronutrients during critical illness
ii) Causes of low micronutrient levels during critical illness
iii) Associations between micronutrients levels and outcome during critical illness
iv) Supplementation of micronutrients during critical illness
v) Micronutrient levels unchanged or high during critical illness

**Fig. 2:**
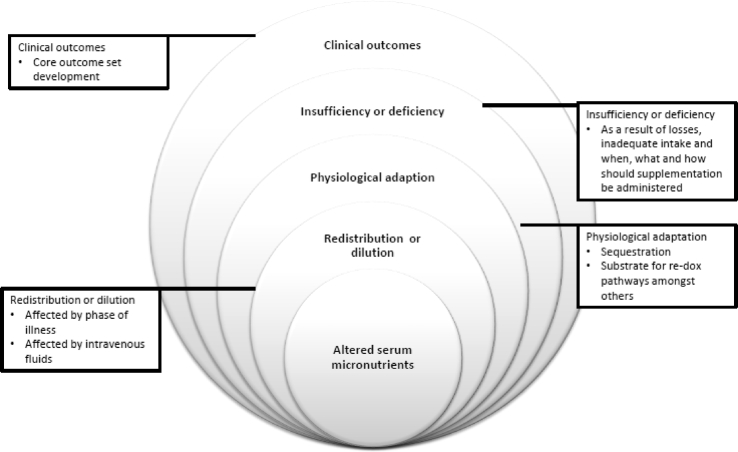
Framework used to characterize concepts of micronutrient status in critically ill children.

These were used to develop a conceptual framework for factors impacting on micronutrient status during critical illness (Fig. 2).

#### 3.1.1 Study characteristics

The studies included in the scoping review examined micronutrient status in 1855 infants and children in 35 studies. All but one study reported serum or plasma levels of vitamins and trace elements. Although ascorbate levels (a vitamin C salt) levels were reportedly low in the cerebrospinal fluid of children with traumatic brain injury (TBI) [30], this was excluded as it was not serum/plasma micronutrient levels. Studies included in the scoping review investigated serum/plasma levels, reporting micronutrient status was determined on admission day in majority of patients. There were a number of studies describing low serum/plasma micronutrient levels during critical illness including thiamine, riboflavin, folate, vitamin B6, vitamin B12, vitamin A, β-carotene, zinc, selenium, iron and chromium [[31], [32], [33], [34], [35], [36], [37], [38], [39], [40], [41]], as well as studies describing serum/plasma levels unchanged or high during critical illness vitamin E, vitamin B6, copper and manganese [38,[42], [43], [44]]. A number of these studies reported factors associated with serum/plasma levels of micronutrients levels including the use of continuous renal replacement therapy, cardiac surgery and systemic inflammatory response [21,22,30,31,37,38,42,[44], [45], [46], [47], [48], [49], [50], [51], [52], [53], [54], [55]]. Associations between micronutrients levels and morbidity e.g. multi-organ failure, lactic acidosis, sepsis and mortality e.g. thiamine deficiency were also reported [40,41,46,53,56]. There were only two studies considering micronutrient supplementation in critically ill children e.g. zinc and whey protein, zinc, glutamine, selenium and metaclopramide [21,22,46] (Table 2, Table 3).

**Table 2:** Characteristics of studies descringing serum/plasma levels of micronutrients during paediatric critical illness.

Title	Authors	Published	Patient characteristic	Time points (day)	Micro- nutrients	Methodology	Aim & results	Conclusion
Vitamin B deficiencies in a critically ill autistic child with a restricted diet	Baird, J. S.	Nutr Clin Pract 2015; 30(1): 100-103	n = 1 critically ill child	0	B vitamins	Case study	A case study describing a children with autism was admitted with hepatomegaly and liver dysfunction, as well as severe lactic acidosis. A diet history revealed his diet self-limited to a single fast food fried chicken. The child was found to be deficient in multiple micronutrients, including the B vitamins thiamine and pyridoxine. Lactic acidosis improved rapidly with thiamine; 2 weeks later, status epilepticus-with low serum pyridoxine-resolved rapidly with pyridoxine.	Micronutrient deficiencies including B vitamin should be considered in critically ill autistic child who presents with a few food diet.
Low serum selenium is associated with the severity of organ failure in critically ill children	Broman M et al.	Clinical Nutrition 2018 Aug;37(4):1399-1405	n = 100 critically ill children	0–5	Selenium	Prospoective cohort study	The aim of this study was to characterise the relationship selenium, glutathione status and multiple organ failure in critically ill children. The concentrations of serum selenium and reduced and total glutathione were determined at admission and at day 5. The results showed that selenium was almost 20% lower in patients with multi-organ failure as compared to patients with zero or single organ failure (p < 0.0001). Low concentration of serum selenium as well as a high-reduced fraction of glutathione (GSH/tGSH) was associated with multiple organ failure (p < 0.001 and p < 0.01) respectively. A correlation between low serum selenium concentration and high-reduced fraction of glutathione (GSH/tGSH) was also seen (r = −0.19 and p = 0.03). The serum selenium concentrations in the pediatric reference group in a selenium poor area were age dependent with lower concentrations in infants as compared to older children (p < 0.001). Almost half of the patients had a PICU-stay >5 days and these patients showed an increase in selenium of 14% from admission to 5 days. Children undergoing treatment with continuous renal replacement therapy (CRRT) showed an increase in selenium over 5 days.	A low serum selenium concentration was associated with the development of multiple organ failure.
The randomized comparative pediatric critical illness stress-induced immune suppression (CRISIS) prevention trial∗∗	Carcillo J.A et al.	Pediatric Critical Care Medicine; Mar 2012; vol. 13 (no. 2); p. 165-173	n = 298 critically ill children, randomised to receive one of 2 nutraceuticals	0–28	Whey protein vs. zinc, selenium, glutamine, metoclopramide (a prolactin secretalogue)	Randomized Controlled Trial	To determine whether nutraceutical supplementation of either whey protein or zinc, selenium, glutamine, metoclopramide (a prolactin secretalogue) reduced the risk of developing nosomial infection or sepsis in critical ill children. Patients were stratified according to immunocompromised status and randomly assigned to receive daily enteral zinc, selenium, glutamine, and intravenous metoclopramide (n = 149), or daily enteral whey protein (n = 144) and intravenous saline for up to 28 days of intensive care unit stay. The primary end point was time to development of nosocomial sepsis/infection. There were no differences by assigned treatment in the overall population with respect to time until the first episode of nosocomial infection/sepsis (median whey protein 13.2 days vs. zinc, selenium, glutamine, and intravenous metoclopramide 12.1 days; p = 0.29 by log-rank test) or the rate of nosocomial infection/sepsis (4.83/100 days whey protein vs. 4.99/100 days zinc, selenium, glutamine, and intravenous metoclopramide; p = 0.81). At baseline 79–89% of patients had low zinc levels and 55–57% had low selenium levels. C-reactive protein levels were not reported in this study.	There was no benefit to providing zinc, selenium, glutamine, and intravenous metoclopramide compared to whey protein.
Interaction Between 2 Nutraceutical Treatments and Host Immune Status in the Pediatric Critical Illness Stress-Induced Immune Suppression (CRISIS) Comparative Effectiveness Trial	Carcillo JA et al.	JPEN Journal of Parenteral & Enteral Nutrition; Nov 2017; vol. 41 (no. 8); p. 1325-1335	n = 298 critically ill children, randomised to receive one of 2 nutraceuticals	0–28	Whey protein vs. zinc, selenium, glutamine, metoclopramide (a prolactin secretalogue)	Randomized Controlled Trial	Posthoc analysis of children enrolled in the CRISIS study (∗∗ further study results from CRISIS study) comparing 3 admission immune status categories: immune competent without lymphopenia, immune competent with lymphopenia, and previously immunocompromised. The comparative effectiveness of either whey protein or zinc, selenium, glutamine, metoclopramide (a prolactin secretalogue) on immune status was evaluated.C-reactive protein levels were not reported in this study.	There were no meaningful differences between the two groups with regards to immune status in those children who received the zinc, selenium, glutamine and metaclopramide intervention compared to whey protein.
Safety and Dose Escalation Study of Intravenous Zinc Supplementation in Pediatric Critical Illness	Cvijanovich N et al.	Journal of Parenteral and Enteral Nutrition; Aug 2016; vol. 40 (no. 6); p. 860-868	n = 24 critically ill children	0–7	Zinc	Randomized Controlled Trial	The aim of this study was to determine a safe dose of intravenous (IV) Zn to restore plasma Zn levels in critically ill children. This was a stepwise dose escalation study of IV Zn supplementation in critically ill children <10 years of age. Patients were sequentially enrolled into 4 dosing groups [1]: no zinc [2], 250 mcg/kg/d ZnSO4 [3], Zn 500 mcg/kg/d ZnSO4, or [4] Zn 750 mcg/kg/d ZnSO4. ZnSO4 was administered 3 times daily for 7 days. Plasma Zn was measured at baseline, end of first ZnSO4 infusion, 1 h postinfusion, and 7 h postinfusion on day 1, then daily through days 2–7. Baseline plasma Zn was low in all patients (mean [SD], 41.8 [16.0] mcg/dL). Plasma Zn increased over the study period in supplemented groups; however, mean plasma Zn in the Zn750 group exceeded the 50th percentile. Plasma Zn was not associated with IL-6, CRP, or lymphocyte subsets among groups. No patient had a study-related adverse event.	IV zinc supplementation at 500 mcg/kg/d restored plasma Zn to near the 50th percentile.
Zinc homeostasis in pediatric critical illness	Cvijanovich N et al.	Pediatric Critical Care Medicine; Jan 2009; vol. 10 (no. 1); p. 29-34	n = 20 critically ill children	0–3	Zinc	Prospoective cohort study	The aim of this study was to investigate the relationship between a decline in plasma zinc concentrations in critically ill children and metallothionein expression. All patients had low zinc levels (mean, 0.43; range, 0.26–0.66 mug/dL) on day 1 and remained low (mean, 0.51; range, 0.26–0.81 mug/dL) on day 3, even when corrected for hypoalbuminemia. In comparison, serum copper levels were normal. On day 1, there was a positive correlation between zinc levels and expression of MT-1A (p < 0.01), MT-1G (p = 0.02), and MT-1H (p = 0.03). Plasma zinc levels correlated inversely with C-reactive protein levels (r = -00.75, p = 0.01) and interleukin-6 levels (r = -0.53, p = 0.04) on day 3. On day 3, patients with two or more organ failures had significantly lower plasma zinc concentrations compared with patients with ≤ 1 organ failure (p = 0.03).	Plasma zinc concentrations are low in critically ill children and is associated with reduced metallothionein expression. Plasma zinc correlated with measures of inflammation (C-reactive protein and interleukin-6) and the degree of organ failure on day 3.
Coma and respiratory failure in a child with severe vitamin B(12) deficiency	Codazzi D et al.	Pediatr Crit Care Med 2005; 6(4): 483–485.	n = 1 critically ill child	0	Vitamin B12	Case study	To describe the neurological sequalae of vitamin B12 deficiency in a 10 month old exclusively breastfed infant of a vegan mother. Chronic dietary vitamin B(12) deprivation was confirmed by blood and urinary samples. Treatment with vitamin B(12) led in 2 wks to rapid and complete hematological improvement and to partial regression of neurologic symptoms. During the following 3 yrs the boy had normal vitamin intake and underwent intensive rehabilitative treatment.	Vitamin deficiency may have long last effecting on clinical outcomes and rapid clinical improvement following deficiency correction does not always correlate with complete recovery. In this case there was an improvement in brain volume, but linguistic and psychomotor delay persisted.
Factors associated with not meeting the recommendations for micronutrient intake in critically ill children.	Dos Reis Santos M et al.	Nutrition 2016; vol. 32 (no. 11–12); p. 1217-1222	n = 260 critically ill children	0–5	Micronutrients	Retrospective cohort study	The aim of this study was to identify factors associated with not meeting dietary recommended intake (DRI) of zinc, selenium, cholecalciferol, and thiamine in enterally fed critically ill children which was compared to estimated average requirement (EAR) and adequate intake (AI) values during the first 10 d of ICU stay. The majority of patients did not meet the recommendations for micronutrients. After adjusting for covariates, age <1 year, malnutrition, congenital heart disease, use of inotropes and renal replacement therapy were associated with failure to meet the recommendations for at least one of the micronutrients studied.	Factors associated with failure to meet the recommendations for micronutrient intake in children receiving enteral tube feeding during an ICU stay are low weight for age, fluid restriction and disease severity.
Baseline serum concentrations of zinc, selenium, and prolactin in critically ill children.	Heidemann, S et al.	Pediatric Critical Care Medicine; May 2013; vol. 14 (no. 4)	n = 235 children admitted to PICU	0–3	Crisis	Prospoective cohort study	The aim of this study was to describe serum concentrations of zinc, selenium, and prolactin in critically ill children within 72 h of PICU admission, in addition to characterising any relationship with lymphopenia. Zinc levels ranged from <0.1 μg/mL to 2.87 μg/mL (mean 0.46 μg/mL and median 0.44 μg/mL) and were below the normal reference range for 235 (83.9%) children. Selenium levels ranged from 26 to 145 ng/mL (mean 75.4 ng/mL and median 74.5 ng/mL) and were below the normal range for 156 (56.1%) children. C-reactive protein levels were not reported in this study.	Serum concentrations of zinc, selenium, and prolactin are often low in critically ill children following admission to PICU.
The impact of cardiopulmonary bypass on selenium status, thyroid function, and oxidative defense in children	Holzer R et al.	Pediatr Cardiol 2004; 25(5): 522–528.	n = 59 critically ill children	0	Selenium	Prospoective cohort study	The objective of this study was to investigate the relationship between plasma selenium and thyroid hormone status in pediatric cardiac surgical patients. There was a significant reduction in the plasma selenium concentration after cardiopulmonary bypass with obtained median measurements of 0.61 μmol/L (induction) and 0.51 μmol/L (48 h postoperatively).	Plasma selenium in children undergoing cardiopulmonary bypass significantly decreases and reduced thyroid function
Low plasma selenium concentrations in critically ill children: the interaction effect between inflammation and selenium deficiency.	Iglesias B et al.	Critical care May 2014; vol. 18 (no. 3); p. R101	n = 173 critically ill children	2–5	Selenium	Prospoective cohort study	The aim of this study was to determine what factors were associated with low plasma selenium in critically ill children. A prospective study was conducted in 173 children (median age 34 months) with systemic inflammatory response who had plasma selenium concentrations assessed 48 h after admission and on the 5th day of ICU stay. The normal reference range was 0.58 μmol/L to 1.6 μmol/L. Malnutrition and CRP were associated with low plasma selenium. The interaction effect between these two variables was significant. When CRP values were less than or equal to 40 mg/L, malnutrition was associated with low plasma selenium levels (odds ratio (OR) = 3.25, 95% confidence interval (CI) 1.39 to 7.63, P = 0.007; OR = 2.98, 95% CI 1.26 to 7.06, P = 0.013; OR = 2.49, 95% CI 1.01 to 6.17, P = 0.049, for CRP = 10, 20 and 40 mg/L, respectively). This effect decreased as CRP concentrations increased and there was loose significance when CRP values were >40 mg/L. Similarly, the effect of CRP on low plasma selenium was significant for well-nourished patients (OR = 1.13; 95% CI 1.06 to 1.22, P < 0.001) but not for the malnourished (OR = 1.03; 95% CI 0.99 to 1.08, P = 0.16). The acute phase response and malnutrition are associated with low plasma selenium.	Plasma concentrations as an index of selenium status is low in patients with acute systemic inflammation.
Effect of blood thiamine concentrations on mortality: Influence of nutritional status.	Leite H, de Lima et al.	Nutrition 2018; vol. 48; p. 105-110	n = 202 critically ill children	0–10	Thiamine	Prospoective cohort study	The aim of this study was to evaluate blood thiamine concentrations in critically ill children. The primary outcome variable was 30-d mortality. Mean blood thiamine concentrations within the first 10 days of ICU stay.Thiamine deficiency was detected in 61 patients within the first 10 d of ICU stay, 57 cases being diagnosed on admission and 4 new cases on the 5th day. C-reactive protein concentration during ICU stay was independently associated with decreased blood thiamine concentrations (p = 0.003). There was a significant statistical interaction between mean blood thiamine concentrations and malnutrition on the risk of 30-d mortality (p = 0.002). In an adjusted analysis, mean blood thiamine concentrations were associated with a decrease in the mortality risk in malnourished patients (odds ratio = 0.85; 95% confidence interval [CI]: 0.73–0.98; P = 0.029), whereas no effect was noted for well-nourished patients (odds ratio: 1.03; 95% CI: 0.94–1.13; P = 0.46).	Low levels of thiamine in malnourished patients was associated with increased risk of 30 day mortality, but not in well nourished patients.
Increased plasma selenium is associated with better outcomes in children with systemic inflammation	Leite, H et al.	Nutrition 2015; 31(3): 485–490.	n = 99 critically ill children	0–5	Selenium	Prospoective cohort study	The aim of this study was to assess changes in plasma selenium levels and outcome of critically ill children. Plasma selenium increased from admission (median 23.4 mug/L, interquartile range 12.0–30.8) to day 5 (median 25.1 mug/L, interquartile range 16.0–39.0; P = 0.018). Following adjustment for confounding factors, a delta selenium increase of 10 mug/L was associated with reductions in ventilator days (1.3 d; 95% confidence interval [CI], 0.2–2.3; P = 0.017) and ICU days (1.4 d; 95% CI, 0.5–2.3; P < 0.01). Delta selenium >0 was associated with decreased 28-d mortality on a univariate model (odds ratio, 0.67; 95% CI, 0.46–0.97; P = 0.036). The mean daily selenium intake (6.82 mug; range 0–48.66 mug) was correlated with the increase in selenium concentrations on day 5.	An increase in plasma selenium is independently associated with shorter times of ventilation and ICU stay in children with systemic inflammation.
Low blood thiamine concentrations in children upon admission to the intensive care unit: risk factors and prognostic significance	Lima L et al.	Am J Clin Nutr 2011; 93(1): 57–61.	n = 202 critically ill children	0	Thiamine	Prospoective cohort study	The aim of this study was to determine the prevalence of and identify factors associated with low blood thiamine concentrations upon admission of children to a pediatric intensive care unit. Low blood thiamine concentrations upon admission were detected in 57 patients (28.2%) and were shown to be independently associated with C-reactive protein concentrations >20 mg/dL (odds ratio: 2.17; 95% CI: 1.13, 4.17; P = 0.02) but not with malnutrition. No significant association was shown between low blood thiamine concentrations upon admission and outcome variables.	The incidence of low blood thiamine concentrations upon admission was high. Only the extent of the systemic inflammatory response showed an independent association with this event.
Lactic acidosis as presenting symptoms of thiamine deficiency in children with haematological malignancy	Lerner R et al.	J Pediatr Intensive Care 2017; 6:132–135	n = 2 children with haematological malignancies	0	Thiamine	Case study	This case report describes haemodynamic instability in two children with haematological malignancies with low thiamine levels which were inversely related to lactate levels. Following thiamine administration the lactic acidosis resolved.	For children with haematological malignancies admitted to PICU with low blood pressure and lactic acidosis should be screened for thiamine deficiency with supplementative given if levels are low.
Assesment of serum zinc, selenium and prolactin	Negm F et al.	Pediatric Health, Medicine and Therapeutics 2016:7 17–23	n = 50 critically ill children	Day 0	Zinc, selenium and prolactin	Prospoective cohort study	The aim of this study was to explore the association of blood Zna d Se levels and immunomodulators in critically ill children. Children who had two organs affected have levels of zinc (median is 56.0 mg/dL) lower than that in patients in whom one organ was affected (median is 82.0 mg/dL). Selenium levels (median is 133.0 ng/mL) were lower in patients in whom one-organ was affected (median is 143.0 ng/mL). Levels of zinc, selenium, and prolactin in patients with sepsis (medians were 77.0 mg/dL, 142.0 ng/mL, and 18.2 ng/mL) were lower than that in patients without sepsis (medians were 81.0 mg/dL, 160.0 ng/mL, and 30.2 ng/mL). Zinc was significantly inversely correlated with organ failure injury (OFI) score (p = 0.047), and PRL was significantly inversely correlated with OFI score (p = 0.049). There was no correlation between selenium and OFI score. Zinc was significantly inversely correlated with PELOD score (P = 0.026), and PRL was significantly inversely correlated with PELOD score (p = 0.039). There was no correlation between selenium and PELOD score.	Serum concentrations of zinc and prolactin were lower in critically ill children and greater in those with organ dysfunction/failure and during sepsis.
Increased plasma selenium is associated with better outcomes in children with systemic inflammation.	Pons Leite H et al.	Nutrition; Mar 2015; 31 (no. 3): 485-490	n = 99 critically ill children	0–5	Selenium	Prospoective cohort study	The aim of this study was to evaluate changes in plasma selenium on the outcome of critically ill children. Plasma selenium was prospectively measured in n = 99 critically ill children from admission until day 5. Selenium was given only as part of enteral diets. Age, malnutrition, red cell glutathione peroxidase-1 activity, serum C-reactive protein, Pediatric Index of Mortality 2, and Pediatric Logistic Organ Dysfunction scores were analyzed as covariates. Plasma selenium concentrations increased from admission (median 23.4 μg/L, interquartile range 12.0–30.8) to day 5 (median 25.1 μg/L, interquartile range 16.0–39.0; P = 0.018). After adjustment for confounding factors, a delta selenium increase of 10 μg/L was associated with reductions in ventilator days (1.3 d; 95% confidence interval [CI], 0.2–2.3; P = 0.017) and ICU days (1.4 d; 95% CI, 0.5–2.3; P < 0.01). Delta selenium >0 was associated with decreased 28-d mortality on a univariate model (odds ratio, 0.67; 95% CI, 0.46–0.97; P = 0.036). There was an association with PIM2, glutathione, malnutrition with ICU free days but not CRP. The mean daily selenium intake (6.82 mg; range 0–48.66 mg) was correlated with the increase in selenium concentrations on day 5. These findings raise the hypothesis that selenium supplementation could be beneficial in children with critical illnesses.	An increase in plasma selenium is independently associated with shorter times of ventilation and ICU stay in children with systemic inflammation.
Low serum zinc level: The relationship with severe pneumonia and survival in critically ill children	Saleh N et al.	International Journal of Clinical Practice; Jun 2018; 72(6)	n = 320 critically ill children	0	Zinc	Prospoective cohort study	The aim of this study was to assess serum zinc levels in children admitted with pneumonia on admission. 320 critically ill children admitted to the paediatric intensive care unit (PICU) with severe pneumonia. Serum zinc measured in all patients on admission. Serum zinc level was significantly lower among patients admitted to PICU compared with patients admitted to wards (P < 0.001). There was a significant decrease in zinc level in critically ill children complicated by sepsis, mechanically ventilated cases and those who died. Regarding the diagnosis of sepsis, zinc had an area under the curve (AUC) of 0.81 while C-reactive protein (CRP) had an AUC of 0.83. Regarding the prognosis, zinc had an AUC of 0.649 for prediction of mortality, whereas the AUC for Pediatric risk of mortality (PRISM), Pediatric index of mortality 2 (PIM2) and CRP were 0.83, 0.82 and 0.78, respectively. The combined zinc with PRISM and PIM2 has increased the sensitivity of zinc for mortality from 86.5% to 94.9%.	Zinc levels on admission were low in critically ill children with pneumonia.
Thiamine, riboflavin, and pyridoxine deficiencies in a population of critically ill children.	Seear M et al.	The Journal of pediatrics; Oct 1992; 121(4):533-538		0–14	Thiamine, riboflavin, pydridoxine	Prospoective cohort study	The aim of this study was to assess tissue stores of the dependent vitamin cofactors for thiamine (vitamin B1), riboflavin (vitamin B2), and pyridoxine (vitamin B6) using activated enzyme assays (erythrocyte transketolase, glutathione reductase, aspartate aminotransferase). B vitamin status of three groups of children [1]: 27 patients who were fed solely by nasogastric tube for more than 6 months [2], 80 children admitted to a pediatric intensive care unit for more than 2 weeks, and [3] 6 children receiving intensive chemotherapy was prospectively evaluated. 10 (12.5%) of 80 patients receiving intensive care and 4 of 6 patients receiving chemotherapy were thiamine deficient. Elevated levels returned to normal after thiamine supplementation. No patients were pyridoxine deficient, but 3 (3.8%) of the 80 patients receiving intensive care and 1 of the 6 patients receiving chemotherapy were also riboflavin deficient.	High risk groups for thiamine deficiency are children who are critically ill or receiving oncology treatment for children cancer.
Thiamine deficiency in children with congenital heart disease before and after corrective surgery	Shamir R et al.	JPEN 2000;24(3): 154–158.	n = 12 critically ill children	0–5	Thiamine, pydridoxine	Prospoective cohort study	To determine whether there was an association with thiamine deficiency treatment and the use of loop diuretics in children with congenital heart disease following cardiac surgery. Overall, 18% (1/12 with VSD and 3/10 with TOF) of children with congenital heart disease had thiamine deficiency before surgery. Three of the four children with TD had adequate intake of thiamine.	Thiamine deficiency is common in children with congenital heart disease, but was not associated with nutritional status or diuretic use.
An unusual cause of persisting hyperlactatemia in a neonate undergoing open heart surgery	Simalti A et al.	World J Pediatr Congenit Heart Surg 2015; 6(1): 130-134	n = 1	0	Thiamine	Case study	A single case of persistent hyperlactaemia in an infant with congenital heart disease. In case of persistently high lactate levels with no other evidence of cellular hypoperfusion, administration of thiamine resulted in symptom resolution.	Symptom resolution with treatment
Thiamine Deficiency Leading to Refractory Lactic Acidosis in a Pediatric Patient	Teagarden A et al.	Case Rep Crit Care 2017: 5121032	n = 1	0	Thiamine	Case study	A term neonate with malignant pertussis required extracorporeal membrane oxygenation and continuous renal replacement therapy, developed profound lactic acidosis of unknown etiology. The patient had thiamine deficiency and the acidosis resolved rapidly with vitamin supplementation.	Symptom resolution with treatment
Multiple Micronutrient Plasma Level Changes Are Related to Oxidative Stress Intensity in Critically Ill Children.	Valla FV et al.	Pediatr Crit Care Med. 2018;19(9): e455-e463	n = 201 critically ill children	0–2	Micronutrients	Prospoective cohort study	The aim of this study was to describe the plasma concentrations of Se, Zn, Cu, vitamin A, vitamin E, vitamin C, and β-carotene in severe oxidative stress conditions in critically ill children, compared with healthy control children. Three groups of patients were defined: severe oxidative stress PICU group (at least two organ dysfunctions), moderate oxidative stress PICU group (single organ dysfunction), and healthy control group (prior to elective surgery); oxidative stress intensity was controlled by measuring plasma levels of glutathione peroxidase and glutathione. Here was a significant trend (p < 0.02) toward plasma level decrease of six micronutrients (selenium, zinc, copper, vitamin E, vitamin C, and β-carotene) while oxidative stress intensity increased.	During critical illness there are multiple micronutrients where deficiency or redistribution occurs with severe oxidative stress.
Prognostic value of blood zinc, iron, and copper levels in critically ill children with pediatric risk of mortality score III.	Wang G et al.	Biological trace element research; Jun 2013; 152 [3]:300-304	n = 31 critically ill children	0	Zinc, iron and copper	Prospoective cohort study	The aim of this study was to explore the association of blood Zn, Fe, and Cu concentrations and changes in the pediatric risk of mortality (PRISM) score in critically ill children. Zn and Fe levels were significantly lower in patients than in controls (p < 0.05). There was no significant difference in Cu levels (p > 0.05). In critically ill children, blood Zn and Fe concentrations were inversely correlated with PRISM III score (Zn: r = −0.36; Fe: r = −0.50, both p < 0.05).	Serious illness in neonates may lead to decreased Zn and Fe blood concentrations.
Blood zinc, iron, and copper levels in critically ill neonates.	Wang G et al.	Biological trace element research; Mar 2015; 164 (1):8-11	n = 46 critically ill children	0	Zinc, iron and copper	Prospoective cohort study	The aim of this study is to explore the prognostic value of blood zinc, iron, and copper levels in critically ill neonates by comparing blood metal levels with the score for neonatal acute physiology (SNAP). Forty-six neonates admitted to the neonatal intensive care unit. Blood Cu, Zn, and Fe values were measured by inductively coupled plasma atomic emission spectrophotometry. Ill neonates were divided into extremely critical (SNAP ≥ 10) and critical groups (1 ≤ SNAP < 9). Zn levels were lower in patients than in controls (p < 0.05). Cu levels did not differ between patients and controls (p > 0.05). Fe levels were not significantly between the critical and control groups (p > 0.05). In ill neonates, blood Zn and Fe concentrations in the extremely critical group were lower than in the critical group (p < 0.05). Serious illness in neonates may lead to decreased Zn and Fe blood concentrations.	Serious illness in neonates may lead to decreased Zn and Fe blood concentrations.
Matched Retrospective Cohort Study of Thiamine to Treat Persistent Hyperlactatemia in Pediatric Septic Shock	Weiss S et al.	Pediatr Crit Care Med. 2019	n = 6 critically ill children	0–3	Thiamine	Case study	To characterise the effect of thiamine on physiologic and clinical outcomes for children with septic shock and hyperlactatemia. Lactate was greater than 5 mmol/L for a median of 39 h (range, 16.1–64.3 h) prior to thiamine administration for cases compared with 3.4 h (range, 0–22.9 h) prior to maximum lactate for controls (p = 0.002). There was no difference in median (interquartile range) change in lactate from T0 to T24 between thiamine-treated cases and controls (−9.0, −17.0 to −5.0 vs −7.2, −9.0 to −5.3 mmol/L, p = 0.78), with both groups exhibiting a rapid decrease in lactate. There were also no differences in secondary outcomes between groups.	Treatment of pediatric septic shock with thiamine was followed by rapid improvement in physiologic and clinical outcomes after prolonged hyperlactatemia.
Continuous renal replacement therapy amino acid, trace metal and folate clearance in critically ill children.	Zappitelli M et al.	Intensive care medicine; 2009; 35 [4]: 698-706	n = 15 critically ill children requiring CVVHD	0–5	Folate and trace metals	Prospoective cohort study	The aim of this study was to prospectively evaluate for 5 days the impact of continuous veno-venous hemodialysis (CVVHD) on amino acid, trace metals and folate clearance in critically ill children prospectively for 5 days. Blood concentrations (amino acids, copper, zinc, manganese, chromium, selenium and folate) were measured at CVVHD initiation, and on days 2 and 5. At CVVHD initiation, serum zinc and copper concentrations were below the reference range, then normal by day 2 and 5. Serum manganese levels were always above the normal range. On day 2 and 5 there was negative balance for selenium, but positive for other trace metals. However, folate concentrations decreased significantly by CVVHD day 5 Folate clearance was 16 mL/min per 1.73 m(2) on Days 2 and 5 and serum concentrations decreased significantly from initiation to Day 5 (p=< 0.05).	The use of CVHHD may impact on micronutrient status in the longer term.
Vitamin A deficiency in critically ill children with sepsis	Zhang X et al.	Pediatric Critical Care; 2019; 23(1): 267	n = 160 critically ill children	Day 0	Vitamin A	Prospoective cohort study	The aim of this study was to characterise the prevalence of vitamin A deficiency in critically ill children with sepsis and clinical outcomes. Vitamin A deficiency was found in 94 (58.8%) subjects in the study group and 6 (12.2%) subjects in the control group (P < 0.001). In septic patients, 28-day mortality and hospital mortality in patients with vitamin A deficiency were not significantly higher than that in patients without vitamin A deficiency (P > 0.05). However, vitamin A levels were inversely associated with higher PRISM scores in septic children with VAD (r = - 0.260, P = 0.012). Vitamin A deficiency was associated with septic shock with an unadjusted odds ratio (OR) of 3.297 (95% confidence interval (CI), 1.169 to 9.300; P = 0.024). In a logistic model, vitamin A deficiency (OR, 4.630; 95% CI, 1.027–20.866; P = 0.046), procalcitonin (OR, 1.029; 95% CI, 1.009–1.048; P = 0.003), and the Pediatric Risk of Mortality scores (OR, 1.132; 95% CI, 1.009–1.228; P = 0.003) were independently associated with septic shock.	The prevalence of vitamin A deficiency was high in children with sepsis.

**Table 3:** Micronutrient function, reference ranges for vitamins, minerals and trace elements [[91], [92], [93], [94]].

Micronutrient	Role	Age	Reference range (US)	Reference range (SI)	Levels reported Median (interquartile range)	Direction of serum change Low No change ≈ High	Studies describing changes in serum levels of micronutrients during critical illness
**Thiamine/Vitamin B1 (p)**	Thiamine is involved in a number of intermediate metabolism associated with energy production including converting pyruvate from glucose into acetyl co-enzyme A for entry into the Krebs cycle, during thiamine deficiency alters intermediate metabolism resulting in lactic acidosis [31].		5.3–7.9 μg/mL		5.5 μg/mL (5.1, 6.5)	Low	Low serum thiamine levels [[32], [33], [34],^36^,^65^] have been reported in 12.5–32% patients [31,42,47]
**Pyridoxine/Vitamin B6 (p)**	Vitamin B6 is required for 150 enzyme reactions, including inflammatory pathways including the kynurenine pathway, sphingosine 1-phosphate metabolism, the transsulfuration pathway, and serine and glycine metabolism [95].		5–50 μg/l		Levels not reported	Low **No change ≈**	A single case report of vitamin B6 (and thiamine deficiency) [36]. Levels were unchanged [42]
**Riboflavin/Vitamin B2 (p)**	Riboflavin acts in synergy with a number of other B vitamin plays a role in energy metabolism and production, in addition to red blood cell formation [96].		5.3–7.9 μg/mL		Levels not reported	Low	3.8% are reported to have a low riboflavin levels [42]
**Folate/Vitamin B9 (s)**	Folate is required for one-carbon transfer reaction, which includes the methylation of lipids, amino acid and deoxyribonucleic acid [97].	Newborn >1 mo	7.0–32 ng/mL 1.8–9.0 ng/mL	15.9–72.4 nmol/l 4.1–20.4 nmol/l	Levels not reported	Low	Low levels of folate [38] were reported in patients on continuous veno-venous filtration (CVVH)
**Vitamin B12 (s)**						Low	A single case reported vitamin B12 deficiency [35]
**Vitamin A (s)**	Vitamin A and β-carotene are required for growth, vision, the immune system and as an antioxidant [98].	0–1 yr 1–11yr 11–16yr 16–19yr	8–53.6 μg/dl 27.5–44.4 μg/dl 24.9–55 μg/dl 28.7–75.1 μg/dl	0–2 μmol/l 1–2 1–2 1–3	1.9 μmol/l (1.9, 1.92)	Low	Vitamin A deficiency in 58.8% of children with septic shock [39] Low β-carotene was reported in critically ill children with oxidative stress [44]
**Ascorbic acid/Vitamin C (s)**	Vitamin C is an electron donor acting as an antioxidant as well as being required for collagen synthesis [99]		26.1–84.6 μmol/l		31.2 μmol/l (23.5, 39)	Low	Low serum vitamin C was reported in critically ill children with oxidative stress [44]
**α-tocopherol/Vitamin E (s)**	Vitamin E is a fat-soluble vitamin with many functions including antioxidant properties [100].	0–1 yr 1-1 yr	0.2–2.1 mg/dl 0.6–1.4 mg/dl	5–50 μmol/l 14.5–30 μmol/l	15.3 μmol/l (13.9, 16.7)	Low **No change ≈**	Low serum vitamin E was reported in critically ill children with oxidative stress [44]serum levels unchanged children [52] or neonates [43]
**Zinc (p)**	Zinc is a trace element involved in numerous functions including anti-oxidant function, and during the acute inflammatory response with zinc redistribution of zinc in tissues involved in protein synthesis and immune cell proliferation which is associated with reduced serum levels [48].		11–24 μmol/l		7.1 μmol/l (4.6, 7.8)	Low	Serum zinc levels during the first few days of admission are low in children [22,50] with severe pneumonia [48,49], sepsis [50] and in those with Iinflammation [44,48,49], oxidative stress [44] and following CRRT [38,80]
**Selenium(s)**	Selenium is a trace element is involved in anti-oxidant, immunological and endocrine pathways, in addition to helping to maintain membrane and assist in thyroid production [50].	<1 year >1 year	45–130 ng/mL 70–150 ng/mL		39.5 ng/mL (5.5, 165)	Low	Serum selenium levels are reported to be low during the first few days of admission [11,22,37,44,50,53,56], sepsis [50] in those with inflammation and oxidative stress [44,56], following CRRT [38,80] and cardiac surgery [54]
**Copper (s)**	Copper is required for redox pathway, energy production, glucose and cholesterol metabolism [101].		12–29 μmol/l		12.1 μmol/l (9.5, 14.6)	Low **No change ≈** High	Low serum copper was reported in critically ill children with oxidative stress [44] Levels unchanged in critical ill children [49] High in children requiring renal replacement therapy [38]
**Iron (p)**	Iron is required for the normal development of red blood cells and cognitive development. Iron deficiency anaemia affects children in particular [102].	All ages	22–184 μg/dl	4–33 μmol/l	3.9 μmol/l (3.8, 4.5)	Low	Serum iron levels were significantly lower in critically ill children [52] and neonates [43] on admission
**Chromium(s)**	Chromium has been suggested to be required for carbohydrate and lipid metabolism by enhancing the effectiveness of insulin [103].		1.4 μg/L		Levels not reported	Low	Low levels of chromium [38] were reported in patients on CVVH
**Manganese (s)**	Manganese is required as a co-enzyme for a number of enzymes, including macronutrient metabolism, bone formation and oxidative function [104].		9–24 nmol/l		Levels not reported	**No change** ≈ **High**	Levels were unchanged in children with oxidative stress [44] High levels in children requiring renal replacement therapy [38]

**Studies describing outcomes associated with micronutrient levels during critical illness**	
Mortality risk	• Low thiamine levels were associated with increased mortality risk in malnourished patients [83]
**Studies describing between micronutrients levels and morbidity during critical illness**	
Inadequate intake	• Low micronutrient intake from enteral feeds were; low weight for age, fluid restriction, disease severity, the use of alpha-adrenergic drugs and renal replacement therapy [55] • Low thiamine levels are associated with malnutrition [42] • Low plasma selenium levels are associated with malnutrition [37] • A case study of child with autism and a severely restricted vegan diet admitted to the PICU was associated with thiamine and vitamin B6 deficiency [36].
Inflammatory response	• Low serum levels of thiamine [31], iron, zinc and selenium [11,37,56] and zinc [48,49] are associated with the magnitude of acute phase inflammatory response
Oxidative stress	• With increasing oxidative stress plasma levels of six micronutrients decreased vitamin A, C, E, β, selenium, copper and zinc [44].
Medication	• Low thiamine levels was not associated with diuretic use [47]
CCRT	• Low levels of selenium, zinc and chromium [38] • Dilution effects of dialysate in renal replacement therapy result in losses of selenium, thiamine, folate, pyridoxine and vitamin C and accumulation of manganese [80].
Cardiac surgery	• In case study reports thiamine deficiency was associated with lactic acidosis in septic shock [32], extracorporeal membrane oxygenation [33], haematological malignancies [65] and following cardiac surgery [34]. Low selenium levels have been described in children requiring cardiopulmonary bypass [54]
Multi-organ failure	• Low plasma selenium levels are associated with multi-organ failure [53]
Lymphopenia	• Low zinc levels were also found in patients with lymphopenia [51]. In case study reports thiamine deficiency was associated with lactic acidosis in septic shock [32], extracorporeal membrane oxygenation [33], haematological malignancies [65] and following cardiac surgery [34].
Severity of illness score	• Low serum iron levels were associated with severity of illness score critically ill children [52] but not in neonates [43]. Serum copper levels were not associated with severity of illness score [43,52]. • Low serum vitamin A levels in children with septic shock are associated with severity of injury scores [39]. • Low plasma selenium levels are associated with severity of risk scores [48,50,52].
Lactic acidosis	• In case study reports thiamine deficiency was associated with lactic acidosis in septic shock [32], extracorporeal membrane oxygenation [33], haematological malignancies [65] and following cardiac surgery [34].
**Studies describing supplementation of micronutrients during critical illness**	
Zinc	• Two studies considered micronutrient supplementation in critically ill children. One considered the safety of intravenous zinc supplementation in children, which was reported as well tolerated but not associated clinical outcomes were reported [46].
Enteral whey, zinc, selenium, metaclopramide	• Critically ill children expected to require >72 h PICU stay were randomised to receive enteral whey, zinc, selenium and metaclopramide, there was no difference reported in the clinical outcomes compared to the placebo [21,22].

(s) = serum, (p) = plasma, (US) United States, (SI) standard international.

## 4 Discussion

**Fig. 3:**
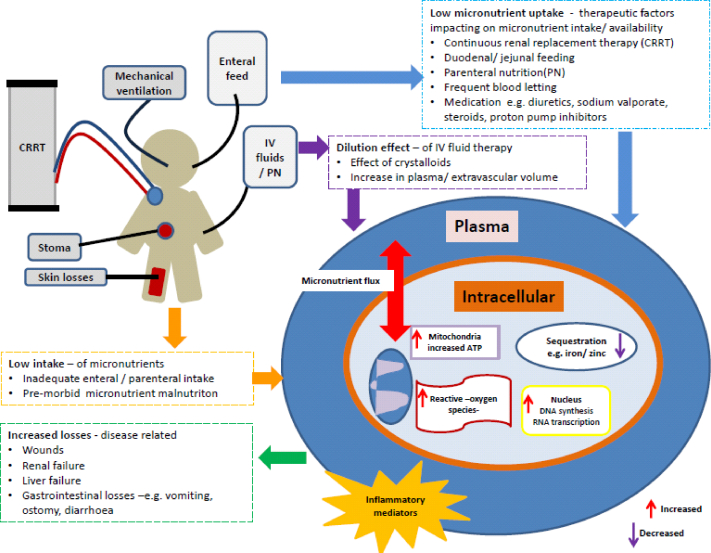
**Schematic of factors impacting on micronutrient status during critical illness**. In the early phase of critical illness, aberrant serum micronutrient levels may be due to 1) redistribution from central circulation to tissues and organs during the acute phase inflammatory response to critical illness, 2) micronutrient losses due to exudative or stomas losses, 3) reduced stores of enzyme co-factors due to increased requirements during illness and 4) low endogenous levels due to pre-existing diseases. Adapted with permission from Casaer M et al. [8].

The results of this scoping review reveals there are numerous gaps in knowledge relating to 1) the interpretation of serum levels of individual micronutrients during critical illness, 2) the causality of associations between micronutrient levels and clinical outcomes, and 3) the impact on clinical outcome with micronutrient supplementation during PICU admission. In the early phase of critical illness, aberrant serum micronutrient levels may be due to 1) redistribution from central circulation to tissues and organs during the acute phase inflammatory response to critical illness, 2) micronutrient losses due to exudative or stomas losses, 3) reduced stores of enzyme co-factors due to increased requirements during illness and 4) low endogenous levels due to pre-existing diseases or dysregulated renal excretion [8,40,41,[57], [58], [59], [60]] (Fig. 3).

Despite the availability of well defined serum/plasma reference ranges, cautious interpretation of low levels is recommended during critical illness, particularly when there is an infection or systemic inflammatory response [11,59]. To counter this effect other quantification methods have been developed including, measuring co-enzyme activity using high performance liquid chromatography [61], and assays to measure erythrocyte concentration [[62], [63], [64]]. Studies of critically ill adults have demonstrated erythrocyte concentration of riboflavin and vitamin B6 status may be a more reliable indicator of micronutrient status when compared to plasma [[62], [63], [64]], although in contrast erythrocyte concentration has not been shown to be a reliable measure of vitamin C and E status [64].

The magnitude of the inflammatory response and subsequent high serum levels of inflammatory mediators (e.g. cytokines) [31] is associated with low serum levels of a number of micronutrients including, thiamine [[31], [32], [33], [34],^36^,^42^,^65^], β-carotene, vitamins A [39], C, E, selenium [37,44,53,56], zinc [44,48,49], copper [44], and iron [52]. Due to the difficulty with regards to interpretering serum/plasma levels during critical illness [11,12], it is not known whether low (or high) serum levels of micronutrients are a reflection of pathophysiological responses to critical illness [11,[66], [67], [68], [69]], a true deficiency state [11], or rather as a markers of illness severity [44].

However, there is limited knowledge regarding the effect of micronutrient supplement on intracellular signalling pathways [70], and as such whether goals of restoring serum levels to within normal range would be of clinical benefit during the acute phase of critical illness. Studies examining factors associated with redistribution of metal ions in response to infection suggest that low levels of extra- and intracellular iron [71,72] and zinc [73] during infection may be a protective mechanisms against pathogens [71,72]. *In vitro* studies of activated macrophages infected with fungal pathogens demonstrate Zn sequestration into intracellular niches or binding to proteins (such as albumin) in order to reduce availability [73]. Several micronutrients are essential for mitochondrial function and acts as cofactors for energy metabolism or anti-oxidant pathways, and inadequate reserves may hinder mitochondrial bioenergetics. However, supplementation may not always be of benefit, and although there are no studies describing vitamin E or C administration on mitochondrial function in critical illness, effect of vitamin E and C supplementation on athletes has been studied, with unexpected results [74]. Athletes who took vitamin C [74] or C and E [75], to reduce the effect of oxidative stress following endurance exercise appeared to have an unintended consequences with reduced mitochondrial bioenergenesis arising from reduced perioxisome proliferator activated receptor gamma co-activator, nuclear respiratory factor and mitochondrial transcription factor A. This prevented exercise induced expression of cytochrome C and reduced the maximal rate of oxygen consumption hampering cellular adaptation to endurance training [74,75].

During critical illness, mobilisation of stores may occur but if there are inadequate stores to meet increased demands during critical illness or the recovery phase, conditional deficiency may occur. Particularly as micronutrient levels available in current enteral or parental nutrition support, are based on RNI for healthy children (Table 4). This has been characterised in a study considering micronutrient intake during paediatric critical illness from enteral feeds. Low micronutrient intake from this route of nutrition support was associated with low weight for age, fluid restriction, diseases severity, the use of alpha-adrenergic drugs and renal replacement therapy [55]. As such, low serum levels, especially during the recovery phase in the absence of systemic inflammation, may therefore be reflective of relative deficiency states particularly in children pre-admission [[76], [77], [78]]. This may also be true for those with increased losses arising from thermal injuries [27], ostomies [79] or from renal replacement therapy [38,80]. Equally low levels may arise as a result of a dilution effects of dialysate or crystalloids used in medical and surgical procedures such as cardio-pulmonary bypass [11,54]. Following a period of critical illness, micronutrients available in enteral feed or parenteral micronutrient mixes may not replete losses during rehabilitation, possibly resulting in growth failure and poor metabolic, physiological and immune resilience [81,82].

**Table 4:** Macro- and micronutrient content of enteral feed per 100 mL compared to reference nutrient intake per day according to WHO and EFSA [[105], [106], [107]].

	Recommended nutrient intake (RNI)					Average macro- and micronutrient per 100 mL^a^				
	RNI <12 mo	RNI 1–3 yrs	RNI 4–6 yrs	RNI 7–10 yrs	RNI11- 18 yrs	Standard infant feed	Energy dense infant feed	Paediatric feed	Paediatric feed 1.5 kcal	Standard adult feed
Energy kcal	545–920	1230	1715	1970	2200	67	100	100	150	102
Protein g	14.9	14.5	19.7	28.3	42.1	1.3	2.6	2.8–3.0	4–4.5	3.3
iron_mg	–	–	–	–	–	0.53	1.2	1	1.5	1.3
zinc_mg	–	–	–	–	–	0.5	0.8	1	1.5	1.1
copper_mg	0.4	1	1	1	1.1–1.3	40	65	81	122	108
manganese_mg	0.02–0.5	0.5	1	1.5	2–3	7.5	0.016	0.15	0.23	0.24
selenium_μg	15	15	20	35	55–70	1.5	2.2	3	4.5	4.9
chromium_ μg	–	–	–	–	–	0	<8	3.5	5.3	5.1
vitamin_a_ μg _re	350	400	400	500	600	54	81	41	61	61
vitamin_e_mg_a-_te	5	100–20	100–20	100–20	100–20	1.1	2.1	1.3	1.9	1.3
thiamine_mg	0.2–0.3	0.5	0.7	0.9	1.1–1.2	50	0.15	0.15	0.23	0.15
riboflavin_mg	0.3–0.4	0.5	0.6	0.9	1–1.3	116	0.2	0.16	0.24	0.16
niacin_mg_ne	2–4	6	8	12	16	430	1.2	1.1	1.7	1.5
vitamin_b6_mg	0.1–0.3	0.5	0.6	1.0	1.2–1.3	40	0.11	0.12	0.18	0.15
folic_acid_ μg	80	160	200	300	400	13	16	15	23	21
vitamin_b12_ μg	0.4–0.5	0.9	1.2	1.8	2.4	0.18	0.3	0.25	0.27	0.24
vitamin_c_mg	25–30	30	30	35	40	9.2	14	10	15	10

a Average of macro & micronutrient from the available infants and nutrition formulas available in Europe.

Some studies have described impaired outcomes associated with low levels of micronutrients. Association studies included low levels of thiamine and an increased risk of mortality in malnourished patients [83]. Other studies described inverse relationship between low levels of vitamin A [39], iron [52] zinc [48,50,52] and severity of injury scores e.g. the higher the severity of injury the lower the serum levels, and multi-organ failure [53]. However, the impact of aberrant levels on short or longer term clinical and nutritional outcomes are not known, as the majority of studies describe measures on admission to intensive care. As such there is a paucity of evidence to suggest any causal link between low serum levels of various micronutrients during the acute phase of critical illness and the impact on clinical outcomes [12].

Only two studies have been completed considering micronutrient supplements, one considering intravenous zinc [46], which was safe to give, and the other as part of the CRISIS trial were enteral zinc, glutamine, whey protein, selenium and metaclopramide were administered. In the CRISIS study there was no difference reported in the clinical outcomes compared to the placebo, however children recruited into the treatment arm had low severity of illness scores who may not have required supplementation [21,22]. Neither study demonstrated any clear benefits with respect to outcomes including reduction in length of hospital stay or survival. Part of the consideration for future trials should be around methodological design including adequately powered studies in addition to appropriate patient recruitment.

Many of paediatric studies completed have small numbers with short duration and therefore make interpretation and comparison of studies challenging [12]. At present there are no specific recommendations for micronutrient supplementation in the acute phase of critical illness [40] and pharmacological use of micronutrients remains controversial due to reports of toxicity [11,84] and potentially unintended consequences as described in adult athletes [74,75]. However, this may not always be the case particularly during the recovery phase of critical illness [38,40,80].

**Fig. 4:**
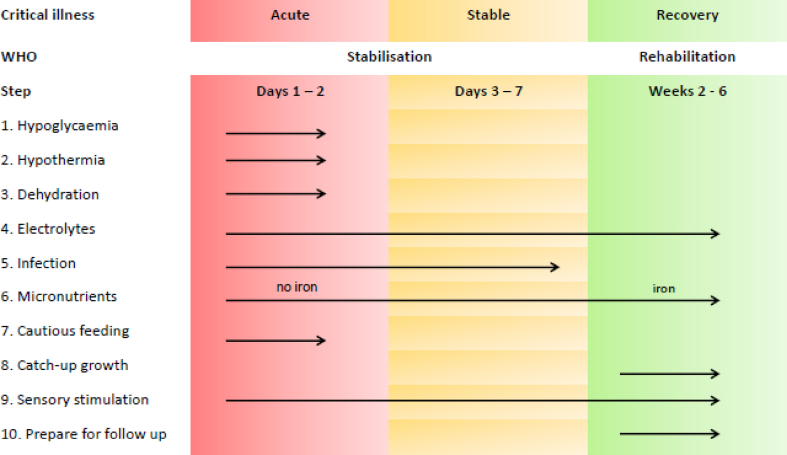
Schematic of World Health Organisation (WHO) recommendations for the management of severe malnutrition. Adapted with permission WHO [86].

With high numbers of critically ill children surviving a period of critical illness [85], nutritional rehabilitation, will require careful consideration. For nutrition rehabilitation to be successful sufficient energy, protein and also micronutrients are required [81,86]. Sub-clinical or clinical micronutrient deficiencies may impede nutritional recovery particularly when anabolism has been restored [77]. Future research should focus on what are the best methods to evaluate micronutrients requirements during critical illness, what micronutrient supplementation (if any) should be provided during critical illness and which cohorts of critically ill children may benefit the most from such supplementation. It may be possible to translate the principles within the World Health Organisation (WHO) recommendations for the management of severe malnutrition, into a conceptual framework for nutrition requirements for critically ill children (Fig. 4) [86].

However, the main challenge for the critical care community is to design research studies which address issues relating to clinical tests of biomarkers using serum or plasma samples which have sufficient sensitivity and specificity, optimal supplementation doses/duration, clinical and nutrition outcome measures [11], particularly as there may be some cohorts of critically ill child for who would benefit from additional micronutrient supplementation. In this scoping review the following factors were identified, which would need to be included within a conceptual framework for determining micronutrient requirements during critical illness (Fig. 2);

1) *micronutrition malnutrition prior to admission* to PICU arising from acute or chronic diseases increased requirement or reduced intake [87,88],
2) *duration/severity of critical illness* and increased micronutrient turnover e.g. oxidative pathways [44].
3) *use of medications or treatment factors impeding absorption* or increasing micronutrient losses and associated transport issues e.g. renal replacement therapy [89],
4) *redistribution, sequestion or dilution* of serum levels of micronutrients and biomarkers to accurately identify the reason for low/high serum values [80],
5) *abnormal* losses of micronutrients as exudates from wounds, drains and stomas impact on micronutrient status [27,90].

At present routine extra supplementation of micronutrients during critical illness to correct low levels in the absence of clear functional pathophysiology as to why levels are low is not recommended [12,40]. However, as survivorship of paediatric critical illness increases ensuring micronutrition malnutrition is addressed to ensure nutritional rehabilitation is achieved post-discharge, is important.

### 4.1 Limitations

This is a scoping review to present the current range of evidence specific to micronutrient status in serum/plasma of critically ill children in young survivors of intensive care. The results should not be generalised beyond this paper other than about the quality of the available evidence. Children with major burns were also excluded from this review as they are not commonly managed within a PICU but a dedicated burns unit, although we acknowledge, they represent a cohort of children with significant nutrition risk [27]. A significant issue within this review was the lack of evidence regarding micronutrient status in critically ill children during the acute phase and into rehabilitation, making it difficult to provide any recommendations. Given this, it was not possible to synthesis results or reliably estimate prevalence and impact of micronutrient status during the acute phase and whether micronutrient supplementation would have any beneficial effect.

## 5 Conclusion

During critical illness, micronutrient should be provided in sufficient amounts to meet reference nutrient intakes for age. Although, there is insufficient data to recommend routine supplementations of micronutrients at higher doses during critical illness, the ‘absence of evidence should not imply evidence of absence’, and well designed prospective studies are urgently needed to elucidate paediatric micronutrient requirements during critical illness. The absence of reliable biomarkers make it challenging to determine whether low serum levels are reflective of a true deficiency or as a result redistribution, particularly during the acute phase of critical illness. As more children continue to survive a PICU admission, particularly those with complex diseases micronutrient supplementation research should also be inclusive of the recovery phase following critical illness.

## Conflict of interest

None.

## Funding

This report is independent research arising from an Integrated Clinical Academic Clinical Lectureship, Luise Marino - ICA-CL-2016-02-001 supported by the National Institute for Health Research and Health Education England. The views expressed in this publication are those of the author(s) and not necessarily those of the NHS, the National Institute for Health Research, Health Education England or the Department of Health and Social Care. SCATV received an unrestricted research grant from 10.13039/501100001720Nutricia Research B.V, The Netherland. The funder had no role in the design and conduct of the study; collection, management, analysis, and interpretation of the data; preparation, review, or approval of the manuscript; and decision to submit the manuscript for publication.

## Contributors statement

Authors made the following contribution to the manuscript [1]: LVM, FVV, SCATV formulated the original idea [2], LVM completed the database search, data extraction and analysis [3] LVM drafted the manuscript [4] LVM, FVV, SCATV, RMB reviewed and revised the manuscript for important intellectual content [5], and all authors provided final approval of the version to be submitted.
